# Correction to: Performance on the Hayling and Brixton tests in older adults: Norms and correlates

**DOI:** 10.1093/arclin/acaf117

**Published:** 2025-12-13

This is a correction to: Allison A.M. Bielak, Laura Mansueti, Esther Strauss, Roger A. Dixon, Performance on the Hayling and Brixton tests in older adults: Norms and correlates, *Archives of Clinical Neuropsychology*, Volume 21, Issue 2, February 2006, Pages 141–149, https://doi.org/10.1016/j.acn.2005.08.006

Table 2 was published with an error for Midpoint age 70 and Category A errors score. The table lists the SD as ``0.01'': this is a typographical error and should read ``1.01''.

The error is outlined in this notice to preserve the Version of Record

